# A budget impact model and a cost–utility analysis of reducer device (Neovasc) in patients with refractory angina

**DOI:** 10.3389/fcvm.2024.1307534

**Published:** 2024-03-18

**Authors:** Agostino Fortunato, Ilaria Valentini, Filippo Rumi, Debora Antonini, Ludovica Siviero, Eugenio Di Brino, Michele Basile, Americo Cicchetti

**Affiliations:** Alta Scuola di Economia e Management dei Sistemi Sanitari (ALTEMS) - Università Cattolica del Sacro Cuore, Roma, Italy

**Keywords:** refractory angina, reducer, budget impact, cost–utility analysis, myocardial ischemia, coronary sinus

## Abstract

**Background:**

Refractory angina (RA) is a chronic condition characterized by the presence of debilitating angina symptoms due to established reversible ischemia in the presence of obstructive coronary artery disease (CAD). Treatments for this condition have undergone major developments in recent decades; however, the treatment for RA remains a challenge for medicine. In this sense, the Coronary Sinus Reducer System (CSRS) stands as the last line of therapy for ineligible patients for revascularization with reversible ischemia. The purpose of this report is to evaluate the potential burden on the National Health Service (NHS) and measure the health effects in terms of both quantity (life years) and quality-of-life aspects related to the reducer.

**Methods:**

Two different economic evaluation models were developed as part of the analysis. The budget impact was developed to estimate the potential burden on the NHS from incremental uptake of the use of the reducer in the target population. The utility cost analysis compares and evaluates the quality of life and health resource use and costs between the two alternatives, based on the research of Gallone et al. A deterministic and probabilistic sensitivity analysis was carried out to characterize the uncertainty around the parameters of the model.

**Results:**

In the budget impact analysis (BIA), the reducer is shown to be more expensive in the first 2 years of the model, due to the gradual uptake in the market and the cost of the device. Starting from the third year, assuming maintenance of effectiveness, there are savings in terms of resource absorption in direct healthcare costs arising from hospitalizations, emergency department accesses, coronarography, and visits avoided.

**Conclusion:**

The BIA and cost-effectiveness model show that the reducer device, despite an increase in resources absorbed in the first years of implementation and use, has the potential to result in increased quality of life in patients with RA. These costs are largely offset in the short term by the improved clinical outcomes achievable leading to savings from the third year onward in the BIA and a dominance ratio in the cost–utility analysis.

## 1 Introduction

Refractory angina (RA), a condition that affects individuals with severe and/or diffuse obstructive coronary artery disease (CAD), presents a persistent burden despite advancements in coronary disease treatments. Approximately 525,000 patients annually in Europe and the United States undergo coronary studies with no indication for revascularization (1). While pharmacological and interventional treatments have significantly extended survival, many patients still suffer debilitating symptoms. According to Giannini et al. (2), a clinical analysis of 141 patients treated with the reducer implant demonstrated a significant improvement in the Canadian Cardiovascular Society (CCS) class and Seattle Angina Questionnaire (SAQ) scores, indicating the safety and efficacy of the device in reducing symptoms and enhancing the quality of life.

In 2019, the guidelines of the European Society of Cardiology (ESC) (3) recommend beta-blockers, calcium channel blockers (CCBs), and additional agents as first- and second-line therapies to manage angina symptoms. However, individualized treatment remains essential due to the lack of definitive evidence favoring any specific antianginal agent in preventing ischemia-related adverse events.

Revascularization procedures such as percutaneous coronary intervention (PCI) or coronary artery bypass graft surgery (CABG) are widespread but not viable for certain patients due to various complexities, leading to the classification of “no-option” patients (4, 5). In this context, increasing blood pressure within the coronary sinus (CS) has emerged as a well-established approach to alleviate RA. The CS Reducer System (CSRS), recently included in the ESC guidelines for chronic coronary syndrome in class IIB (3), addresses this need by elevating CS pressure using an implanted device (6). Furthermore, reducer implantation appears to positively impact left ventricular function according to the study conducted by Tzanis et al. (7), demonstrating significant improvements in left ventricular ejection fraction and volumes, particularly in patients with reduced ejection fraction. The need for wider acceptance of the device within the interventional community has been highlighted in studies by Gallone et al. (8), emphasizing the importance of accurate imaging techniques to fully understand the potential of the device. Moreover, the reducer decreases the ischemic burden, providing a strong physiological base underlying its clinical efficacy, throughout the blood redistribution mechanism, occurring from less ischemic segments to more ischemic ones, within the same layer, rather than with a by-layer pattern (9).

The prevalence of RA is rising due to increasing CAD cases, multiple comorbidities, and an aging population, significantly impacting the quality of life of patients. This leads to frequent hospitalizations and a high level of resource utilization, which is represented by the highest number of sick leave among patients who are still actively working (10). The American College of Cardiology (AAC) and the American Heart Association (AHA) estimated that refractory angina pectoris (RAP) amounts to annual costs in the tens of billions in the United States due to high cost to society, both in terms of healthcare spending and productivity losses (11).

The burden of cardiovascular disease in general is a major cause of health loss in all regions of the world. According to the Global Burden of Disease 2015, an estimated 442.7 million prevalent cases of cardiovascular disease were present worldwide causing an estimated 17.92 million deaths (12). Ischemic heart disease (of which PA is a part) was the leading cause of all declines in quality of life globally and in every region of the world (12).

The purpose of the following article was to evaluate the economic profile of the reducer device. First, a budget impact analysis (BIA) was developed, to estimate the potential burden on the Italian National Health Service (NHS) from incremental diffusion of the use of the Neovasc Reducer System in patients with RAP. Second, a cost–utility model based on a probabilistic decision tree was also developed to compare the cost-effectiveness profile in patients who undergo reducer implantation compared with those treated with “standard of care” (SoC) and evaluate its impact in terms of the incremental cost-effectiveness ratio (ICER). Due to the absence of a defined standard of care in the Italian context for “no-option” patients, SoC group patients refer to the period going from the date of diagnosis of RA to the implant, coherent with the analysis of Gallone et al. (13).

## 2 Materials and methods

Two economic evaluations were developed to estimate the economic impact related to the increased use of the reducer device (Neovasc Inc.) in the Italian care setting. The analysis includes a budget impact and a cost–utility model in patients undergoing reducer implantation compared to those defined as “no option” with the standard of care intended as the period going from condition diagnosis to the implant. For both evaluations, we are considering a 5-year time horizon, with the analyses exclusively taking into account the perspective of the Italian National Health Service (SSN).

### 2.1 Efficacy data

In this section, we are presenting an overview of the main studies investigating the efficacy of the reducer device. These studies offer a comprehensive insight into the performance of the device in treating patients with RA. The COSIRA trial (14), a phase 2 multicenter randomized double-blind sham-controlled clinical trial, evaluated the efficacy and safety of a CS-reducing device in patients with RA who were not candidates for revascularization. The study included 104 patients, randomly assigned to either device implantation or a sham procedure. The primary endpoint was the improvement of at least two CCS angina classes at 6 months post-procedure. The results showed significant improvement in symptoms and quality of life in the treatment group compared to the control group, with 35% of patients in the treatment group achieving the primary endpoint vs. 15% in the control group. There were no significant differences in exercise time or wall motion index changes between the groups. The trial demonstrated that the CS-reducing device significantly improved angina symptoms and quality of life, suggesting its potential as a treatment option for patients with RA (14). Ponticelli et al. (15) conducted a single-center prospective observational analysis in 2019, including 50 RA patients undergoing reducer implantation (15). The mean follow-up was 748 ± 84 days (approximately 2 years). The study assessed the long-term safety and efficacy, focusing on improvements in CCS class and SAQ scores. The key outcomes revealed a significant reduction in angina symptoms and sustained quality-of-life improvements, with no device-related complications. A portion of patients required further coronary interventions, indicating that while the reducer effectively alleviates symptoms, it does not halt coronary disease progression (16). The REDUCER-I study is a multicenter, non-randomized observational study assessing the safety and efficacy of the CS reducer in patients with RA. Patients with CCS class II–IV RA underwent reducer implantation, with outcomes evaluated at baseline, 6 months, and annually for up to 5 years. The primary efficacy endpoint was the reduction in CCS grade at 6 months, and safety endpoints included procedural or device-related major adverse cardiac events (MACE). The results from the first 228 patients showed a 99% procedural success rate, with significant improvements in angina severity and quality of life sustained up to 2 years (16). Moreover, the RESOURCE study is a retrospective observational registry evaluating the CSR in patients with RA across 20 centers in Europe, the United Kingdom, and Israel, involving 658 patients. The study investigated the amelioration of anginal symptoms using the CCS score, procedural success and complications, and MAC as endpoints. With a median follow-up of 502 days, it reported a significant improvement in angina symptoms, with 39.7% of patients achieving a ≥2 CCS class improvement and a procedural success rate of 96.7%. The median follow-up in the RESOURCE study (17) was 502 days. Konigstein et al. (17) conducted a prospective single-arm registry in 2021 involving 99 patients, which examined the long-term effects (>2 years) of CSR implantation in patients with RA. With a median follow-up of 3.38 years, it reported no procedure-related complications, a significant improvement in CCS class from baseline to 1 year (maintained through to the last follow-up), and outcomes indicating safety and sustained efficacy of CSR for angina relief over time. In addition, Hochstadt et al. (18) published a meta-analysis evaluating the effectiveness of CSR in treating RA. It synthesized data from nine studies, including 846 patients, with follow-up periods ranging from 4 months to 2 years. The primary outcome focused on the proportion of patients improving by at least one CCS angina class, with significant improvements reported. The secondary outcomes included procedural success, periprocedural complications, and improvements in SAQ scores and 6-min walk test distances. The results indicated a high procedural success rate, significant symptoms, and quality-of-life improvements (18).

Finally, Silvis et al. (19) coordinated a multicenter investigation evaluating the safety and efficacy of CSR in Dutch patients with RA over 5 years. It involved 132 patients, focusing on the primary efficacy endpoint of CCS class improvement between baseline and 6-month follow-up and the primary safety endpoint of successful CSR implantation without device-related events. The results indicated significant improvement in CCS class for most patients, with successful implantation in 99% of cases and minor complications reported, underscoring the potential of CSR as a safe and effective treatment for RA. The significant improvement in the CCS class observed at the 6-month follow-up was sustained over the long term, demonstrating the potential of CSR for durable symptom relief and improved quality of life in this patient population. The high success rate of implantation and the low incidence of complications further underscored its safety and effectiveness as a long-term treatment option (19). The evidence across multiple studies consistently demonstrates the efficacy of CSR in treating RA. The key findings include significant improvements in CCS angina classes, potentially sustained over periods of up to 5 years, high procedural success rates, and minimal complications.

### 2.2 Model structure

#### 2.2.1 BIA

BIA is used to estimate the likely change in expenditure to a specific budget holder resulting from a decision to reimburse a new healthcare intervention or some other change in policy at an aggregate population level. The budget (or financial) impact is usually calculated using a budget impact model, for 3–5 years, at a national level or for more local healthcare payers and providers. In contrast to cost-effectiveness analyses, which are used to estimate value for money, analyses using budget impact models assess affordability. Two scenarios are usually compared: a world in which the new intervention or policy is implemented and a counterfactual world without the new intervention. Each scenario considers the population size, patient eligibility, speed of uptake, market share of the intervention, and many of the inputs associated with a model-based cost-effectiveness analysis. Budget impact models are commonly used by local- or national-level decision-makers for planning purposes, especially where (extra) expenditure in one budget is offset by savings in another (20).

The model considers a time horizon of 5 years and an Italian initial population in the age group of 65–74 years of 6,795,374 men and women. The prevalence of chronic ischemic heart disease and stable angina is about 30,000 cases per million population (3.3% in men and 3.9% in women). Thus, there are about 245,771 patients with angina pectoris, with a slightly higher distribution for women. Among them, 29,493 (12%) are those suffering from RA pectoris. Based on an expert opinion, it is estimated that approximately 60% of these individuals are eligible to undergo the reducer system intervention. Thus, the target population is equal to 17,696. Table 1 shows the number of eligible patients identified over the time horizon considered by the analysis considering the incidence of the condition.

**Table 1:** Patient funnel.

6.795.374	Italian population 65–74 years
245.771	Population affected by angina pectoris
29.493	Population affected by angina pectoris refractory^a^
17.696	Eligible population to the reducer

^a^ Assumptions provided by expert opinion.

The model simulates two market scenarios. The first scenario depicts the current market conditions, assuming a steady utilization of the device under examination. In contrast, the second scenario envisions a future state where an annual increase of 0.35% in the adoption of the reducer is projected over the 5-year period of the analysis. The comparison between the current market mix and the revised market mix is shown in Table 2. To ensure a consistent comparison between the two scenarios across years, we considered the following drivers: hospitalizations, outpatient visits, coronary angiographies, elective coronary angioplasties, and emergency department accesses.

**Table 2:** Market shares.

Current market mix					
	Y1	Y2	Y3	Y4	Y5
Reducer	1%^a^	1%	1%	1%	1%
Standard of care (SoC)	99%	99%	99%	99%	99%
	100%	100%	100%	100%	100%
Revised market mix					
	Y1	Y2	Y3	Y4	Y5
Reducer	1.5%	1.85%	2.20%	2.55%	2.90%
Standard of care (SoC)	98.5%	98.2%	97.8%	97.5%	97.1%
	100%	100%	100%	100%	100%

^a^ Assumptions provided by the distributor of the device in Italy.

#### 2.2.2 Cost–utility analysis

The cost–utility analysis was conducted to valorize the costs and outcomes associated with patients who undergo the reducer implantation compared to those within the standard of care (SoC) period among adults aged 65 years, according to the Italian NHS perspective. The analysis refers to the hypothetical cohort of 10,000 patients assigned to the two alternatives compared (viz., reducer and SoC).

The cost-effectiveness study was developed using the quality-adjusted life years (QALYs) as an indicator to determine the quality of life deriving from the successfulness or failure of the reducer implant in the model, and the expenditure for healthcare resources is expressed in monetary values. Both the costs and the outcomes were discounted using a discount factor of 3.5% although the time horizon of the analysis is not “lifetime” but takes into consideration a 5-year time horizon. To construct the decision tree model and estimate event probabilities, we mapped out the disease progression in terms of distinct health states (alive/dead) and the potential transitions between these states. This involved detailing the costs and QALYs associated with each state. The cost factors identified align with those utilized in the BIA.

**Figure 1:**
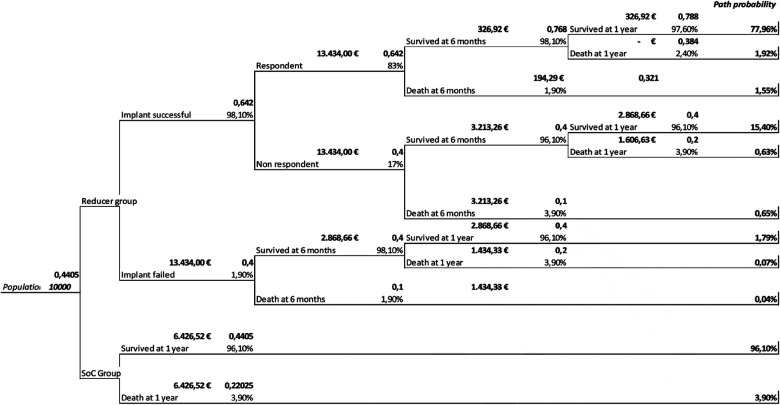
Decision tree model.

In our analysis, we adopted a decision tree model that was previously employed in a study cited in the literature (13), with its structure illustrated in Figure 1. This model, as described in the 2020 study by Gallone et al., was adapted to serve as a conceptual framework for our cost-effectiveness analysis. Transition probabilities between health states (alive/dead) were calculated using Kaplan–Meier estimates derived from the 2020 study by Gallone et al.

The decision tree includes the probabilities, QALYs, and the average costs structured with two branches to allow the comparison of the two groups: reducer and SoC. In the reducer group, the tree branches are based on the success or failure of the system implant. Because of successful implantation, improvement in quality of life resulting from the improved clinical outcomes achievable following implantation of the device is observed.

Costs and QALYs between groups were compared. The cost-effectiveness of the reducer was expressed as an ICER, defined as the differential in the cumulative costs relative to the difference in cumulative QALYs of the reducer and SoC. Contrary to the assumptions made by Gallone et al. (13), which anticipated a decline in effectiveness over time, our analysis supports the sustained and consistent efficacy of the device, as previously documented in the efficacy section and corroborated by literature (14–17). For this reason, a decrease in effectiveness over the time horizon considered is not assumed in the model. The cost-effectiveness ratio of the reducer was evaluated over various time horizons from the moment of implantation, exploring the hypothesis of a constant duration of the effectiveness of the reducer both in terms of clinical outcomes and in terms of QALYs.

### 2.3 Cost inputs

In Italy, the cost of hospital care accounts for 45.5% of the total health expenditure, the second highest cost in percentage terms among the European Union (EU) countries. Interestingly, while total hospital health expenditure has decreased over the years, that generated by patients over 64 years of age is increasing both in absolute numbers and as a percentage (21, 22). The base case considers the following cost inputs. Tariffs of specialist outpatient services and diagnosis-related group (DRG) lists were considered for the evaluation of cost drivers. In addition, some drivers were extrapolated from the paper by Gallone et al., which represents the only economic evaluation to our knowledge developed in the Italian healthcare context. Table 3 shows the cost inputs used in the BIA and cost–utility analysis. To measure the average therapy for patients with RA pectoris, we considered the following: beta-blockers (atenolol, bisoprolol metoprolol aristo), CCBs, nitrates, ivabradine, and ranolazine.

**Table 3:** Cost inputs.

Inputs	Cost
Reducer implant	€7,000.00
Elective PCI	€6,434.00
Hospitalization	€1,870.00 (13, 23)
Outpatient visit	€88.06
ED admission	€193.00
Coronarography	€2,142.00

The utilization rates were extrapolated from the study by Gallone et al. (13), and to calculate the baseline, therapy reduction for patients implanted with the reducer was not taken into account. The mean baseline therapy reported in the study by Gallone et al. shows that out of 215 patients, 78.2% RA pectoris patients use beta-blockers (atenolol, bisoprolol metoprolol aristo), 54.5% CCBs, 66.5% nitrates, 18.1% ivabradine, and 31.9% ranolazine [number of anti-ischemic drugs, median (IQR) 3 (2–3)]. In terms of economics, the average annual therapy per patient is €386.44 and was calculated using the average annual cost of each drug, i.e., €27.29 for beta-blockers, €114.04 for CCBs, €37.41 for nitrates, €78.19 for ivabradine, and €827.32 for ranolazines, considering the utilization rates. To estimate the average cost of medical therapy in patients with RA, we referred to AIFA transparency lists and summaries of product characteristics for each pharmaceutical class to identify an average dosage. Table 4 shows the estimates to calculate the average cost of therapy.

**Table 4:** Average cost of therapies.

Drugs	mg/day	Daily costs	Therapy usage	Cost/year	Usage percentage	Distribution
Atenolol	100	€0.13	365			
Bisoprolol	5	€0.09	365	€27.9	78.2%	Beta
Metoprolol aristo	150	€0.15	365			Beta
Calcium channel blockers	5	€0.51	365	€114.04	54.5%	Beta
Nitrates				€37.41	66.5%	Beta
Ivabradine	5	€0.35	365	€78.19	18.1%	Beta
Ranolazine	1,000	€3.74	365	€827.32	31.9%	Beta
Total	€386.44					

### 2.4 Utility

The quality of life (QoL) used for the cost–utility analysis was derived from the literature. Gallone et al. carried out a retrospective, observational, multicenter international study including 215 patients affected by severe RA at eight medical centers in Belgium, the Netherlands, and Italy, where reducer implantation was conducted. The QoL between the reducer and the SoC was directly from patients prior to the reducer implant and from the last face-to-face clinical follow-up, using the SAQ or the EuroQOL health status instrument (EQ-5D) depending on the choice of center. The weight of the utility varies in a range of 0–1: when the score is close to 1, the health is better; in the case of death, the utility is zero.

To measure the effectiveness of the treatment, we considered the drivers including specialist visits, emergency room accesses, hospitalizations, coronarography, and PCI.

In this latter study, clinical outcome data were available for 321.2 patients per year of the SoC period (9 months; interquartile range, 3–24) and 519.2 patients per year of follow-up after reducer implantation (15 months; interquartile range, 8–23). Overall, considering the two periods, SoC and reducer occurred in 1.3 and 0.2 hospitalizations, respectively, for angina per patient per year (3.4 and 1.0 total hospitalization days per patient per year), 0.2 and 0.1 ED admissions for angina per patient per year, 1.0 and 0.2 coronary angiographies for angina per patient per year, 0.3 and 0.1 PCI for angina per patient per year, and 2.1 and 0.7 outpatient visits for angina per patient-year.

A reduction was observed also at 6 months on clinical outcomes: hospitalization for angina per patient per year 1.0 (0.0–2.0) vs. 0.0 (0.0–0.5), *P* < 0.001 (total hospitalization days per patient per year 3.6 (1.3–7.8) vs. 0.0 (0.0–1.7), *P* < 0.001); ED hospitalizations for angina per patient per year 0.0 (0.0–0.0) vs. 0.0 (0.0–0.0), *P* = not significant (NS); coronary angiographies for angina per patient per year 0.8 (0.3–1.5) vs. 0.0 (0.0–0.0), *P* < 0.001; PCI for angina per patient per year 0.0 (0.0–0.4) vs. 0.0 (0.0–0.0), *P* = 0.029; and specialist outpatient visits for angina per patient per year 2.1 (1.1–3.3) vs. 1.3 (0.4–2.3), *P* < 0.001. These reductions in clinical outcome outcomes translated into a significant reduction in associated costs from all three perspectives of the health systems referenced in the study by Gallone et al. Specifically, Table 5 shows the parameters considered by the base case in the budget impact assessment model.

**Table 5:** Efficacy inputs.

	Reducer	Distribution	Standard of care (SoC)	Distribution
Hospitalization	1	Normal	3.4	Normal
ED admission	0.1	Normal	0.2	Normal
Coronarography	0.2	Normal	1	Normal
Outpatient visit	0.7	Normal	2.1	Normal
PCI	0.1	Normal	0.3	Normal

## 3 Results

### 3.1 BIM

The analysis is shown as a differential analysis between the two scenarios (the first one involving a constant share of reducer device use and a second one instead where an incremental uptake is expected over the 5 years of analysis). This comparison distinctly illustrates that the escalated deployment of the reducer within the Italian healthcare context correlates with resource savings starting from the third year of the analysis. Notably, these savings are attributed to the enhancement in health-related outcomes that the device potentially delivers.

Table S1 shows the costs for the first scenario. In this scenario, the resource absorption over the considered time horizon of 5 years is €649,094,680.51, with an average annual impact of €129,818,936.10.

Table S2 shows the costs related to Scenario 2 revised market mix. Under this scenario, the resource absorption over the considered 5-year time horizon is €649,029,741.47, with an average annual impact of €129,805,948.29.

**Figure 2:**
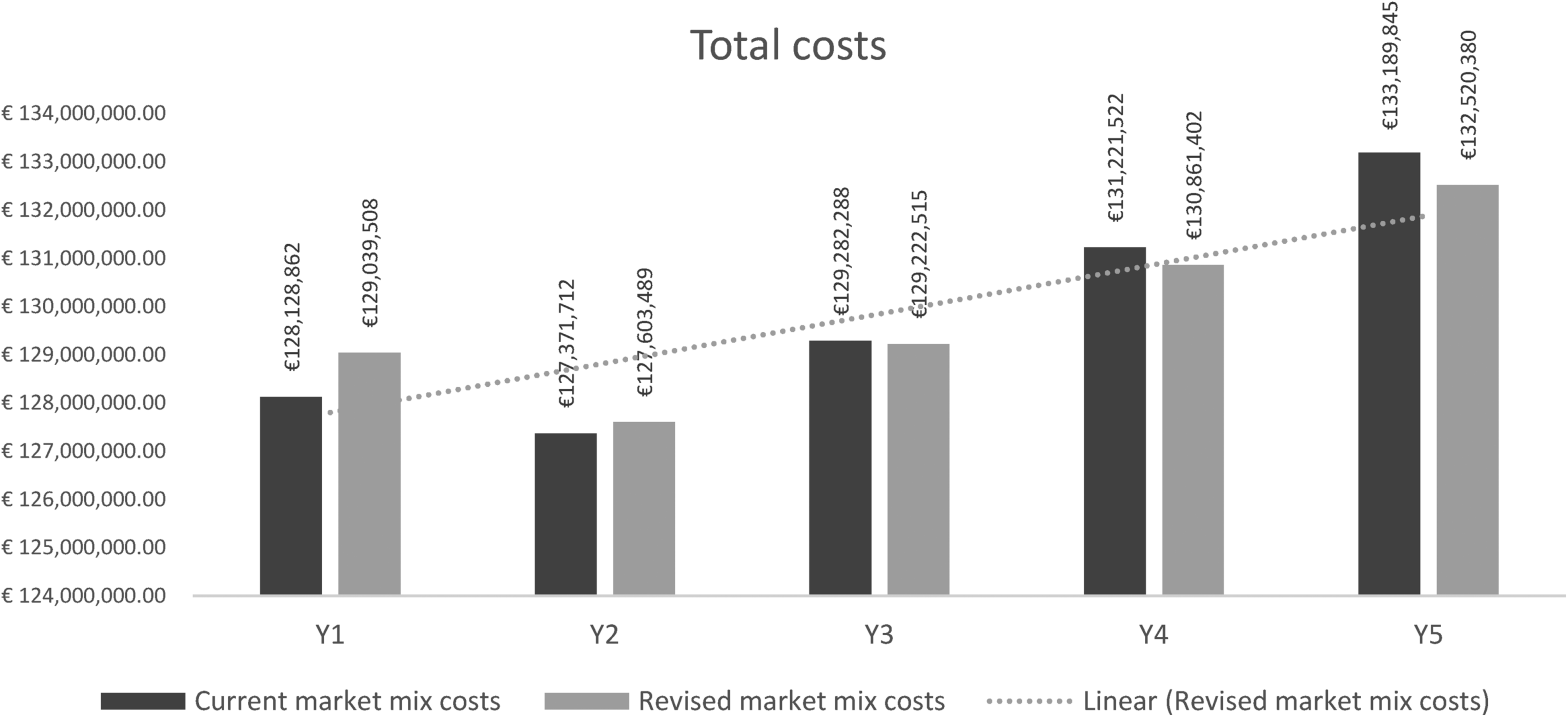
Comparison between Scenario 1 and Scenario 2.

**Figure 3:**
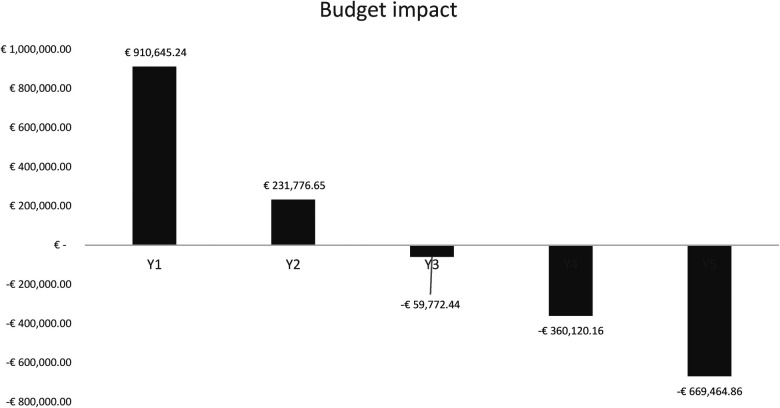
Budget impact.

As depicted in Figure 2, the costs associated with the device are not compensated by improved patient outcomes during the first 2 years of the specified time frame. However, assuming a steady increase in the adoption of the reducer device within the target population, significant savings in terms of resource consumption and direct healthcare costs become evident from the third year onward. This is attributed to decreases in hospitalizations, emergency department admissions, coronary angiographies, outpatient visits, and additional PCI procedures. Figure 3 shows the monetary values resulting from the comparison of the two scenarios under analysis. In Scenario 1 current market mix, the total resource absorption in the first year of analysis is €128,112,243.81, whereas that associated with the alternative scenario (revised market mix) is higher and amounts to €129,014,579.79. The total costs are notably higher in the second year of the time frame; however, by the third year, the savings begin to be modestly realized, and from the fourth year onward, the benefits of resource savings become apparent.

Figure 3 illustrates the outcome of the BIA, revealing incremental resource savings beginning in the third year, amounting to a total of €59,772.44 over the time horizon. The initial 2 years display a diminishing financial burden on the NHS. Savings attributable to the adoption of the reducer device commence in the third year of analysis, with notable reductions in the fourth year (−€360,120.16) and further significant savings in the fifth and final year (−€663,464.86) of the assessed period.

**Figure 4:**
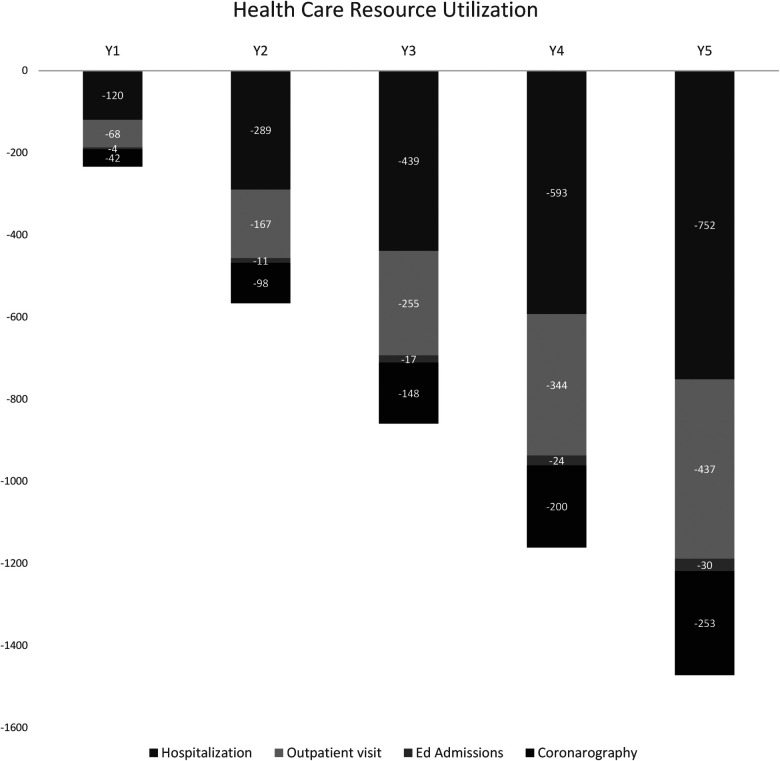
Healthcare resources utilization resulting from the differential analysis of the two scenarios.

The results are shown in Figure 4 regarding the reduction in consumed healthcare resources (hospitalizations, specialist visits, emergency room accesses, coronary examinations, and PCI) resulting from the differential analysis of the two scenarios. There is an incremental reduction in all studied clinical resource utilization variables, considering the time horizon under consideration equal to 5 years. In the last year considered by the model, several avoided hospitalizations of 752 are estimated, implying a total saving of €1,770,737.85. Regarding visits, the incremental deployment of the device would be able to avoid 437 outpatient visits, with €111,924.26, 30 emergency room visits, and 253 coronarographies [non-cumulative healthcare resource utilization (HRU)]. The savings from avoided PCI procedures from the comparison of the two scenarios result in a total (cumulative value over the 5 years) of €165,760,393.94.

### 3.2 CUA

In the study conducted by Gallone et al., 1 year from the baseline, both QALYs and costs were observed to be higher in the reducer group compared to the control (QALY, 0.594 vs. 0.456; *P* < 0.001; costs, €15,702 vs. €6,988 in Italy). This led to an ICER for the reducer implant relative to the SoC treatment of €63,146 per QALY gained in Italy. In our analysis, the ICER at Year 1 in the base case is calculated to be €61,618.60 per QALY. The slight discrepancy is attributed to the decision tree structure accounting for the proportion of patients who do not respond to the implant. In the study by Gallone et al. (13), it is assumed that after the first year, the effect of reducer on costs and utilities would decrease by 30%/year, and despite this decrease, the ICER turns out to be €23,641/QALY at Year 2, while a dominance relationship is inferred at Year 3 (the reducer device turns out to be less expensive and more effective than the “SoC”). In our study, by contrast, the results of the cost–utility analysis showed that between Year 1 and Year 2, the decrease in ICER was 70.56%, dropping from €61,618.60 to €18,143.06. Starting from Year 3 onward, ICER has emerged to be dominant, as shown in Table 6.

**Table 6:** Cost–utility results.

Time horizon: 1 year			
	160,162,439.23 €	75,774,679.70 €	84,387,759.52 €
	5,864.285023	4,389.205	1,475.080023
ICER			57,208.94 €
Time horizon: 2 years			
	184,788,818.12 €	148,594,146.90 €	36,194,671.22 €
	11,477.90628	8,778.41	2,699.496279
ICER			13,407.94 €
Time horizon: 3 years			
	208,820,142.94 €	218,573,654.87 €	−9,753,511.94 €
	16,912.89844	12,061.76362	4,851.134818
ICER			Dominant
Time horizon: 4 years			
	265,281,165.31 €	285,823,962.04 €	−20,542,796.73 €
	22,175.277	15,217.06645	6,958.210545
ICER			Dominant
Time horizon: 5 years			
	288,174,976.93 €	350,451,507.22 €	−62,276,530.29 €
	27,270.84969	18,424.78965	8,846.060046
ICER			Dominant

### 3.3 Sensitivity analyses

#### 3.3.1 Deterministic budget impact model

**Figure 5:**
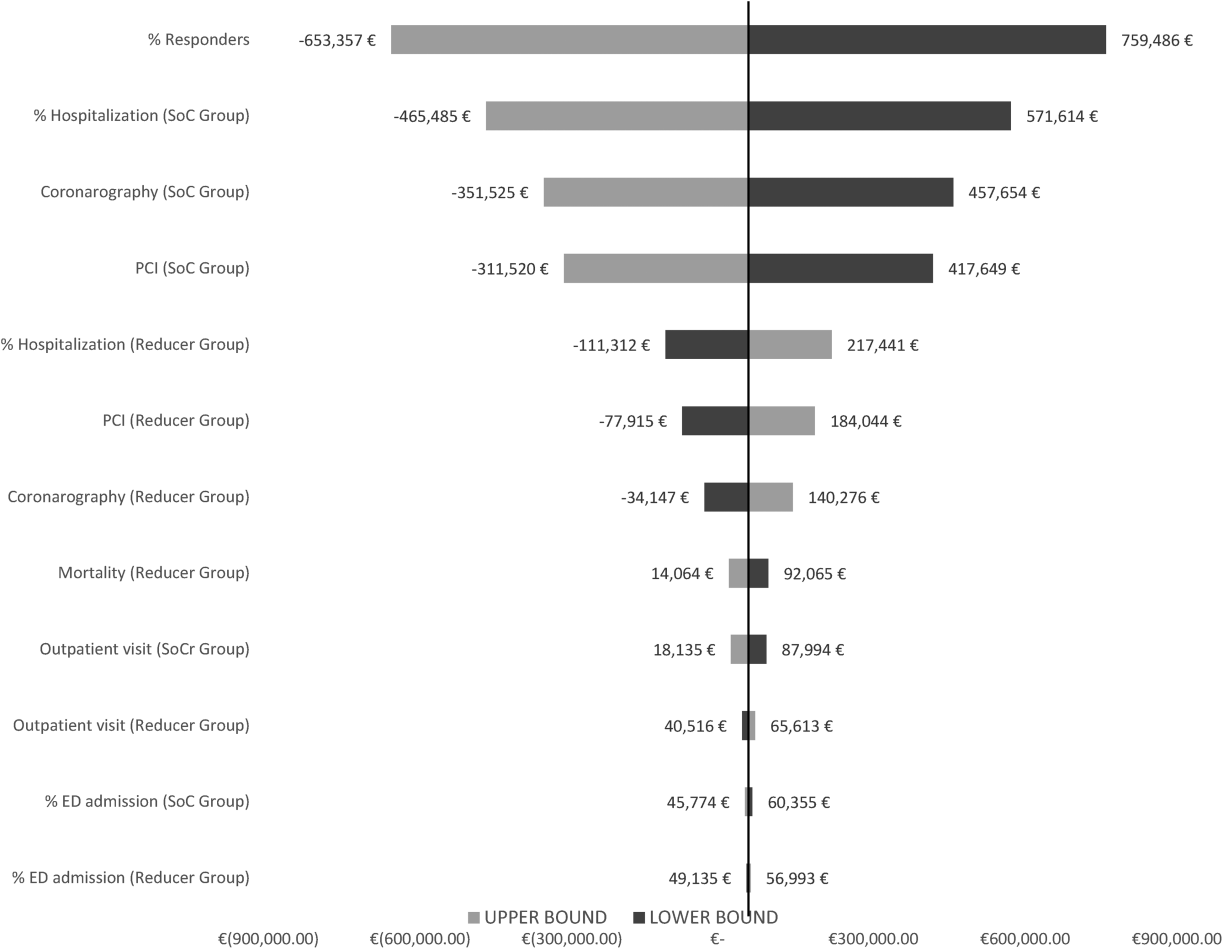
Deterministic sensitivity analysis.

To characterize the uncertainty of the parameters used in the budget impact model, we conducted a deterministic sensitivity analysis, represented using a tornado chart shown in Figure 5. Specifically, the analysis investigates the impact of the results resulting from a predetermined deviation (equal to ±15%) in the parameters of interest. As shown in Figure 5, the parameter that results in a greater deviation from the case base results is the percentage of respondents to the reducer procedure. Among the other parameters characterized by greater uncertainty, we find the number of PCIs the incidence of hospitalizations and coronarographies in Scenario 1 for SoC. Parameters marked by a lower degree of uncertainty, i.e., those parameters whose deviation does not have a significant impact in terms of the results of the analysis, are represented by the incidence of emergency room admissions, mortality rate, reduction in drug therapy, and finally the number of visits.

#### 3.3.2 Probabilistic CUA

**Figure 6:**
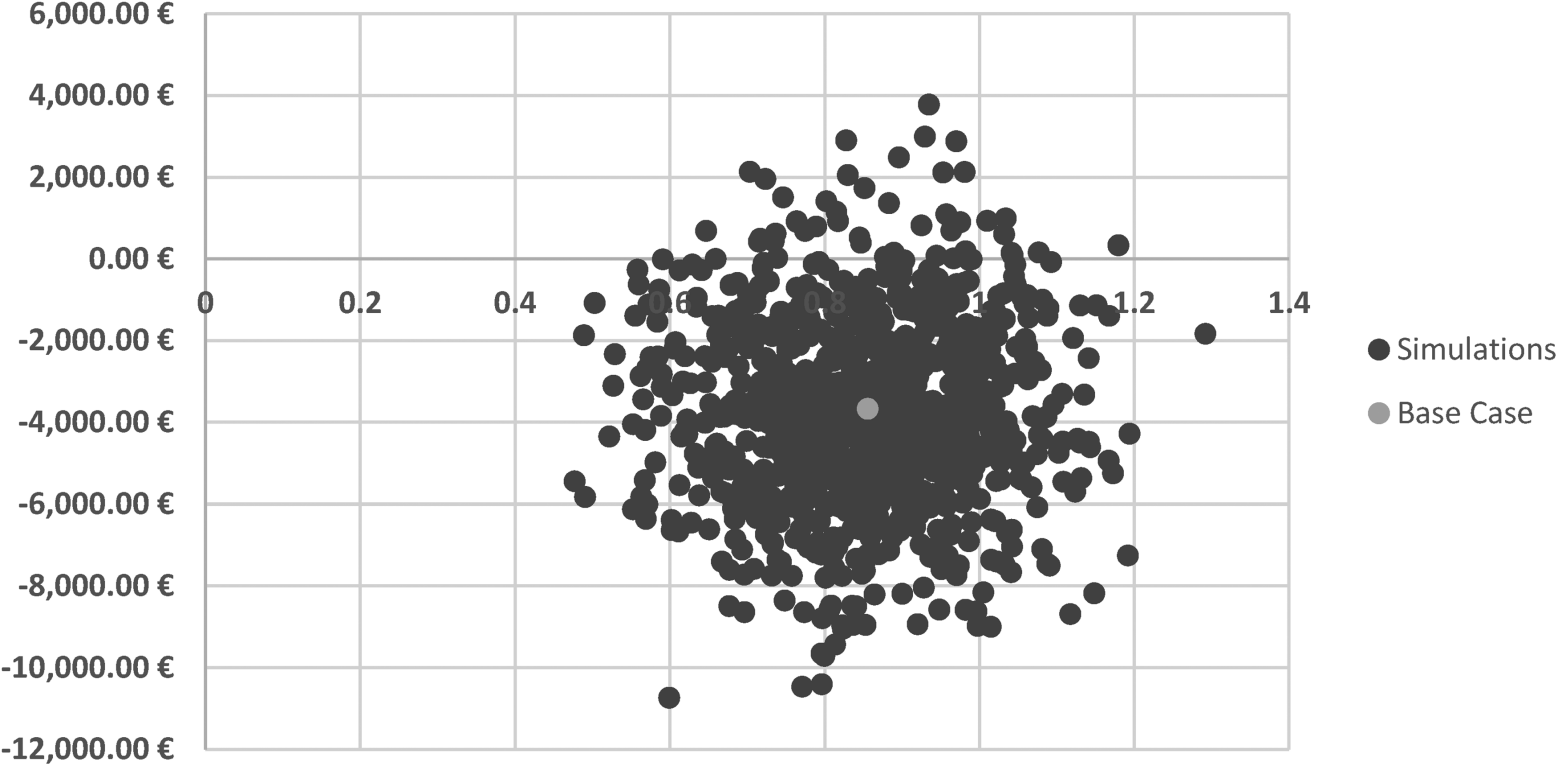
Probabilistic sensitivity analysis.

A probabilistic sensitivity analysis was also carried out on the ICER considering the 5-year time horizon to verify the robustness of the results as the value of the parameters considered for estimating the case base scenario outcome changed. Specifically, a level of uncertainty equal to 15% of the average value of each parameter was considered, each according to a given probability distribution (normal for frequency parameters, beta for probabilities and utilities). A total of 1,000 Monte Carlo simulations were carried out. The results of the probabilistic sensitivity analysis were represented in terms of the deviation of the ICER on the cost-effectiveness plane (Figure 6). As can be seen in Figure 6, most of the simulations fall in the range of dominance with lower average costs for the strategy considering the use of the reducer device, offset by its higher effectiveness in terms of reduction in HRU and QALYs gained compared to the standard of care.

## 4 Limitations

While this study holds significant relevance for its insights into the cost-effectiveness of introducing new technologies within the Italian national healthcare system, it is not without limitations. In contrast to the findings presented by Gallone et al. in 2019 (13), subsequent research (14–17, 19, 21) has suggested that the efficacy of the reducer may remain stable over an extended period. Additionally, the implantation of the reducer has been shown not to affect blood pressure and heart rate metrics pre and post-procedure, further supporting the notion that its beneficial impacts are directly linked to the device itself rather than alterations in medical treatment. Consequently, the reducer emerges as a viable and efficacious option for the majority of patients suffering from RA; however, a potential limitation of our analysis could be the absence of consideration for any decrease in effectiveness over the time horizon. No placebo bias was considered in the present study. In the meta-analysis conducted by Gallone et al. (24) to quantify the impact of placebo on endpoints of symptoms, quality of life, and functional outcomes in randomized placebo-controlled trials (RCTs) of symptomatic stable CAD, a significant placebo effect was observed for all secondary outcomes with overall high heterogeneity. In addition, a substantial effect in placebo-controlled patients emerged in several functional activities and quality-of-life metrics. The high variability emerged to be unjustified by study-related differences or patient characteristics.

Finally, although we have integrated the structure of the decision tree from the study by Gallone et al., this does not mean that the structure correctly reflects all the states and probabilities characteristic of the condition under study. The decision tree is different since it considers the possibility of the implant to be successful or unsuccessful (e.g., patients with coronary microvascular dysfunction). The reducer is clinically utilized for RA patients with significant epicardial CAD with no target for further coronary revascularization. However, a relevant number of patients with stable CAD are sustained by coronary microvascular dysfunction for which the reducer has not been demonstrated to improve symptoms. Indeed, in the sensitivity analysis developed, the first driver whose variance impacts the results the most is the % of responders, which underscores a large uncertainty or ineffectiveness of the device with respect to symptom improvement in the subgroup of patients with coronary microvascular dysfunction.

## 5 Discussion

Optimal medical therapy and advances in coronary/surgical revascularization have improved the survival of patients with ischemic heart disease, shifting the focus to controlling their symptoms and improving their quality of life. In the last years, multiple significant studies have supported the implantation of the reducer in the CS. This latter represents a novel therapeutic option with established safety and efficacy benefits for patients affected by RAP, possibly achieved by enhancing the perfusion of myocardial ischemic territories (25). This is particularly important because RAP patients have no other clinically validated alternatives. As presented in this study, delays in chosen treatment potentially lead to poorer patient outcomes and higher HRU-related costs.

New evidence has been recently published both regarding the device mechanism of action (MOA) and the long-term results: Palmisano et al. showed a significant increase in perfusion and reduction of ischemia with MRI, which addresses both MOA and impact on ischemia; REDUCER 1 (17) study and FIM 12 (26) study include a mean follow-up of 3.38 years and provide evidence that results can be maintained over time. Moreover, the reducer improves myocardial longitudinal and circumferential strain, without microstructural remodeling and impact on diastolic proprieties.

Further studies can confirm the positive long-term results currently published for REDUCER and for clearly understanding the reduction in myocardial ischemia, especially with the advent of physiology measurements (such as IMR and absolute flow). This BIA and cost-effectiveness model demonstrate that, despite initial increases in resource utilization during the early years of implementation and use, the reducer device has the potential to enhance the quality of life for patients with RA. These initial costs are largely offset in the short to medium term by the improved clinical outcomes achieved, leading to cost savings from the third year onward in the BIA and a dominance profile in the cost–utility analysis. By the second year of the cost–utility analysis, the reducer exhibits a favorable cost-effectiveness profile, especially considering that the typical threshold in the Italian context is estimated to be around €30,000–€40,000 per QALY. Further research is required to provide more robust evidence on the economic viability of the reducer within the NHS framework, particularly concerning its long-term effectiveness.

## 6 Conclusion

This analysis determined, by conducting an economic profile assessment, the sustainability of the reducer associated with both increased use of the device over the years of the time horizon under analysis and the cost–utility of the reducer compared with standard of care and to evaluate its impact in terms of ICER, in the management of patients with refractory chronic angina. The results showed that, assuming a constant incremental share of the reducer device uptake in the target population, from the third year onward, device costs are offset by improved clinical outcomes of patients. Specifically, it is noted that the savings from its use over the 5-year period are €39,644.27, with hospitalizations being the most reduced adverse event with 168 avoided hospitalizations. As for the cost–utility analysis, a dominant relationship emerged, compared with the comparator, starting from the same year of the BIA. The following study based its analysis on the model of Gallone et al., expanding the time horizon of the analysis and hypothesizing the possibility of device failure. Furthermore, through some studies found in the literature, it was possible to consider a constant effectiveness in the long term. Further studies will be needed to determine both economic sustainability in the context of the NHS and especially the efficacy in the long term. Considering what emerged during the state of emergency due to COVID-19 and the difficulties to date still demonstrated in the NHS, a device that potentially improves health outcomes in a fragile population by potentially avoiding hospitalizations, visits, and other interventions certainly has an added benefit in view of the organizational impact and can relieve pressure on the NHS processes. Should subsequent extensive and rigorous long-term studies validate the efficacy of the device in question, the findings derived from this analysis stand poised to furnish invaluable insights for decision-makers. Such insights would play a pivotal role in refining and enhancing the strategic allocation of resources and the overall management of patients affected by RA pectoris.

## Data Availability

The original contributions presented in the study are included in the article/Supplementary Material; further inquiries can be directed to the corresponding author.
