# Supplementary material for: A budget impact model and a cost–utility analysis of reducer device (Neovasc) in patients with refractory angina
Source: Front Cardiovasc Med. 2024 Mar 18;11:1307534. doi: 10.3389/fcvm.2024.1307534 (PMC10982321; doi:10.3389/fcvm.2024.1307534)
Supplement: Supplementary file 1 [file Table1.docx]

*Table S1 – Current market mix*

| Current market mix | | | | | | |
| --- | --- | --- | --- | --- | --- | --- |
|  |  |  |  |  |  |  |
| Patients | **Y1** | **Y2** | **Y3** | **Y4** | **Y5** | **Total** |
| Reducer | 177 | 180 | 182 | 185 | 188 | 912 |
| SoC | 17519 | 17781 | 18048 | 18319 | 18594 | 90260 |
| Mortality |  |  |  |  |  |  |
| Patients | **Y1** | **Y2** | **Y3** | **Y4** | **Y5** | **Total** |
| Reducer | 173 | 175 | 178 | 181 | 183 | 890 |
| SoC | 16864 | 17088 | 17345 | 17605 | 17869 | 86770 |
| Management costs (procedures) |  |  |  |  |  |  |
| Reducer Impant | € 2.320.164,85 | € 34.802,47 | € 35.324,51 | € 35.854,38 | € 36.392,19 | € 2.462.538,40 |
| Responders | 143 | 2 | 2 | 2 | 2 | 152 |
| Non responders | 29 | 0 | 0 | 0 | 0 | 31 |
| PCI |  |  |  |  |  |  |
| Reducer | € 92.230,09 | € 112.504,05 | € 114.191,61 | € 115.904,48 | € 117.643,05 | € 552.473,27 |
| SoC | € 32.606.733,87 | € 32.984.702,06 | € 33.479.472,60 | € 33.981.664,68 | € 34.491.389,65 | € 167.543.962,87 |
| Healthcare costs |  |  |  |  |  |  |
| Hospitalization |  |  |  |  |  |  |
| Reducer | € 15.746,33 | € 141.189,60 | € 143.307,45 | € 145.457,06 | € 147.638,92 | € 693.339,36 |
| SoC | € 46.376.668,07 | € 46.914.253,51 | € 47.617.967,31 | € 48.332.236,82 | € 49.057.220,37 | € 238.298.346,08 |
| Outpatient visit |  |  |  |  |  |  |
| Reducer | € 8.836,26 | € 10.778,64 | € 10.940,32 | € 11.104,42 | € 11.270,99 | € 52.930,61 |
| SoC | € 3.123.942,01 | € 3.160.153,88 | € 3.207.556,19 | € 3.255.669,53 | € 3.304.504,57 | € 16.051.826,17 |
|  |  |  |  |  |  |  |
| ED Admission |  |  |  |  |  |  |
| Reducer | € 2.766,62 | € 3.374,77 | € 3.425,39 | € 3.476,77 | € 3.528,93 | € 16.572,48 |
| SoC | € 652.067,11 | € 659.625,69 | € 669.520,07 | € 679.562,87 | € 689.756,32 | € 3.350.532,05 |
| Diagnostic testing |  |  |  |  |  |  |
| Coronarography |  |  |  |  |  |  |
| Reducer | € 61.410,28 | € 74.909,44 | € 76.033,08 | € 77.173,58 | € 78.331,18 | € 367.857,55 |
| SoC | € 36.184.656,49 | € 36.604.098,97 | € 37.153.160,45 | € 37.710.457,86 | € 38.276.114,72 | €185.928.488,49 |
| Medical therapy |  |  |  |  |  |  |
| Reducer | € 55.395,13 | € 67.572,05 | € 68.585,63 | € 69.614,41 | € 70.658,63 | € 331.825,84 |
| SoC | € 6.528.072,55 | € 6.603.744,15 | € 6.702.800,31 | € 6.803.342,32 | € 6.905.392,45 | € 33.543.351,77 |
| Total (NHS Perspective) | **€ 128.128.862,35** | **€ 127.371.711,85** | **€ 129.282.287,53** | **€ 131.221.521,85** | **€ 133.189.844,67** | **€ 649.194.228,26** |

*Table S2 – Revised market mix*

| Revised market mix | | | | | | |
| --- | --- | --- | --- | --- | --- | --- |
|  |  |  |  |  |  |  |
| Patients | **Y1** | **Y2** | **Y3** | **Y4** | **Y5** | **Total** |
| Reducer | 265 | 332 | 401 | 472 | 545 | 2015 |
| SoC | 17430 | 17629 | 17829 | 18032 | 18237 | 89157 |
| Mortality |  |  |  |  |  |  |
| Patients | **Y1** | **Y2** | **Y3** | **Y4** | **Y5** | **Total** |
| Reducer | 259 | 324 | 391 | 461 | 532 | 1967 |
| SoC | 16793 | 16952 | 17145 | 17340 | 17537 | 85767 |
| Management costs (procedures) |  |  |  |  |  |  |
| Reducer Impant | € 3.480.247,27 | € 876.442,27 | € 901.952,48 | € 928.030,80 | € 954.688,53 | € 7.141.361,36 |
| Responders | 215 | 54 | 56 | 57 | 59 | 441 |
| Non responders | 44 | 11 | 11 | 12 | 12 | 90 |
| PCI |  |  |  |  |  |  |
| Reducer | € 138.345,14 | € 201.520,81 | € 244.510,68 | € 288.744,91 | € 334.251,15 | € 1.207.372,70 |
| SoC | € 32.498.181,09 | € 32.741.827,82 | € 33.115.191,65 | € 33.492.389,48 | € 33.873.452,32 | € 165.721.042,35 |
|  |  |  |  |  |  |  |
| Healthcare costs |  |  |  |  |  |  |
| Hospitalization |  |  |  |  |  |  |
| Reducer | € 173.619,50 | € 252.903,29 | € 306.854,45 | € 362.367,23 | € 419.476,36 | € 1.515.220,83 |
| SoC | € 46.222.273,07 | € 46.568.812,64 | € 47.099.849,28 | € 47.636.339,03 | € 48.178.326,01 | € 235.705.600,04 |
| Outpatient visit |  |  |  |  |  |  |
| Reducer | € 13.254,38 | € 19.307,03 | € 23.425,75 | € 27.663,68 | € 32.023,48 | € 115.674,34 |
| SoC | € 3.113.541,93 | € 3.136.884,91 | € 3.172.655,65 | € 3.208.793,71 | € 3.245.302,06 | € 15.877.178,26 |
| ED Admission |  |  |  |  |  |  |
| Reducer | € 4.149,92 | € 6.045,00 | € 7.334,56 | € 8.661,45 | € 10.026,50 | € 36.217,43 |
| SoC | € 649.896,27 | € 654.768,70 | € 662.235,21 | € 669.778,38 | € 677.398,85 | € 3.314.077,42 |
| Diagnostic testing |  |  |  |  |  |  |
| Coronarography |  |  |  |  |  |  |
| Reducer | € 92.115,41 | € 134.180,16 | € 162.804,44 | € 192.257,26 | € 222.557,03 | € 803.914,30 |
| SoC | € 36.064.192,25 | € 36.334.574,24 | € 36.748.907,11 | € 37.167.494,69 | € 37.590.371,40 | € 183.905.539,69 |
| Medical therapy |  |  |  |  |  |  |
| Reducer | € 83.092,69 | € 121.037,19 | € 146.857,72 | € 173.425,62 | € 200.757,53 | € 725.170,75 |
| SoC | € 6.506.339,60 | € 6.555.119,20 | € 6.629.868,98 | € 6.705.386,35 | € 6.781.677,52 | € 33.178.391,65 |
| Total (NHS Perspective) | **€ 129.039.507,60** | **€ 127.603.488,51** | **€ 129.222.515,09** | **€ 130.861.401,69** | **€ 132.520.379,82** | **€ 649.247.292,70** |
